# Molecular Strategies for Intensity-Dependent Olfactory Processing in *Caenorhabditis elegans*

**DOI:** 10.3389/fnmol.2021.748214

**Published:** 2021-11-04

**Authors:** Hankui Cheng, Yu Liu, Yadan Xue, Jiajie Shao, Zhibing Tan, Siyan Liu, Shumin Duan, Lijun Kang

**Affiliations:** ^1^Department of Neurobiology, The First Affiliated Hospital, Zhejiang University School of Medicine, Hangzhou, China; ^2^Department of Neurosurgery, The First Affiliated Hospital, Zhejiang University School of Medicine, Hangzhou, China; ^3^NHC and CAMS Key Laboratory of Medical Neurobiology, MOE Frontier Science Center for Brain Research and Brain-Machine Integration, School of Brain Science and Brain Medicine, Zhejiang University, Hangzhou, China; ^4^Department of Neurology, The Second Affiliated Hospital, Zhejiang University School of Medicine, Hangzhou, China

**Keywords:** olfactory processing, cGMP, intensity dependence, sensory neuron, calcium imaging assay

## Abstract

Various odorants trigger complex animal behaviors across species in both quality- and quantity-dependent manners. However, how the intensity of olfactory input is encoded remains largely unknown. Here we report that isoamyl alcohol (IAA) induces bi-directional currents through a Gα- guanylate cyclase (GC)- cGMP signaling pathway in *Caenorhabditis elegans* olfactory neuron amphid wing “C” cell (AWC), while two opposite cGMP signaling pathways are responsible for odor-sensing in olfactory neuron amphid wing “B” cell (AWB): (1) a depolarizing Gα (GPA-3)- phosphodiesterase (PDE) – cGMP pathway which can be activated by low concentrations of isoamyl alcohol (IAA), and (2) a hyperpolarizing Gα (ODR-3)- GC- cGMP pathway sensing high concentrations of IAA. Besides, IAA induces Gα (ODR-3)-TRPV(OSM-9)-dependent currents in amphid wing “A” cell (AWA) and amphid neuron “H” cell with single ciliated sensory ending (ASH) neurons with different thresholds. Our results demonstrate that an elaborate combination of multiple signaling machineries encode the intensity of olfactory input, shedding light on understanding the molecular strategies on sensory transduction.

## Introduction

Animals sense numerous volatile compounds from environmental cues to locate food sources, avoid predators and pathogens, and communicate with each other (Bargmann, 2006; DeMaria and Ngai, 2010; Wilson, 2013). For example, humans can discriminate more than one trillion odor stimuli (Bushdid et al., 2014). The effects of one given odorant can be influenced by many factors including intensity, context and experience (Bargmann, 2006; Gottfried, 2009; Li and Liberles, 2015; Harris et al., 2019; Duan et al., 2020). However, it remains poorly understood how intensity of odorant signals are encoded by the olfactory system in higher organisms because of the complexity of their nervous systems.

The nematode *Caenorhabditis elegans* offers a powerful model to mechanistically study how the nervous system responds to various odorants at the molecular, cellular and circuit levels. Equipped with a compact nervous system with only 302 neurons and 56 glial cells, *C. elegans* can sense a vast number of odors and execute a wide range of olfactory-related behaviors such as chemotaxis to food sources, pathogen avoidance, social feeding, olfactory associativelearning and imprinting (de Bono and Maricq, 2005; Bargmann, 2006; Chalasani et al., 2007; Ha et al., 2010; Ohno et al., 2014; Duan et al., 2020). Five pairs of sensory neurons have been identified as the main olfactory receptor neurons in *C. elegans*- amphid wing “A” cell (AWA), amphid wing “C” cell (AWC), amphid wing “B” cell (AWB), amphid neuron “H” cell with single ciliated sensory ending (ASH), and amphid neuron “L” cell with dual ciliated sensory endings (ADL) (Bargmann, 2006). AWC and AWA are neurons mainly mediating chemotaxis to food attractants, while AWB, ASH, and ADL are neurons for repellents avoidance (Bargmann, 2006; Harris et al., 2014).

In vertebrates, a large group of G protein-coupled receptors (GPCRs) are employed as olfactory receptors (ORs) (DeMaria and Ngai, 2010). Various odorants bind to distinct ORs and activate the downstream G protein-dependent signal transductions, which eventually open the cAMP-gated CNG channels and Ca^2+^-activated Cl^–^ channels (DeMaria and Ngai, 2010). In *Drosophila*, ORs form odorant-gated ion channels, which allow direct depolarization upon odorant binding (Wilson, 2013). Previous studies have identified a number of genes including GPCRs, Gi/o-like G proteins, cGMP-gated CNG channel (TAX-2/TAX-4), TRP channel (OSM-9) and voltage-gated calcium channel (EGL-19) that are involved in odorants detection and olfactory-related behaviors in *C. elegans* (Brenner, 1974; Bargmann et al., 1993; Bargmann and Kaplan, 1998; Bargmann, 2006; Chalasani et al., 2007; Harris et al., 2014; Ohno et al., 2014; Duan et al., 2020). Notably, a glial cell ensheathing the sensory cilia of olfactory neurons also responds to odorants cell-autonomously and drives olfactory adaptation in *C. elegans* (Duan et al., 2020).

Interestingly, similar to vertebrates, *C. elegans* exhibits distinct behaviors to certain odorants depending on their concentrations (Yoshida et al., 2012; Mainland et al., 2014). Attractive odorants such as IAA, benzaldehyde, 2,4,5-trimethylthiazole and 2,3-pentanedione become aversive at higher concentrations (Yoshida et al., 2012; Duan et al., 2020; Supplementary Figure 1). However, the molecular mechanisms underlying intensity-dependent olfactory transduction remain largely unknown. By integrating genetic manipulation, electrophysiology and calcium imaging at single neuron resolution, here we systematically identified the molecular strategies by which *C. elegans* encodes the intensity of odorant stimulus.

## Results

### Odorants Induce Bi-Directional Currents in Amphid Wing “C” Cell Neurons

**FIGURE 1:**
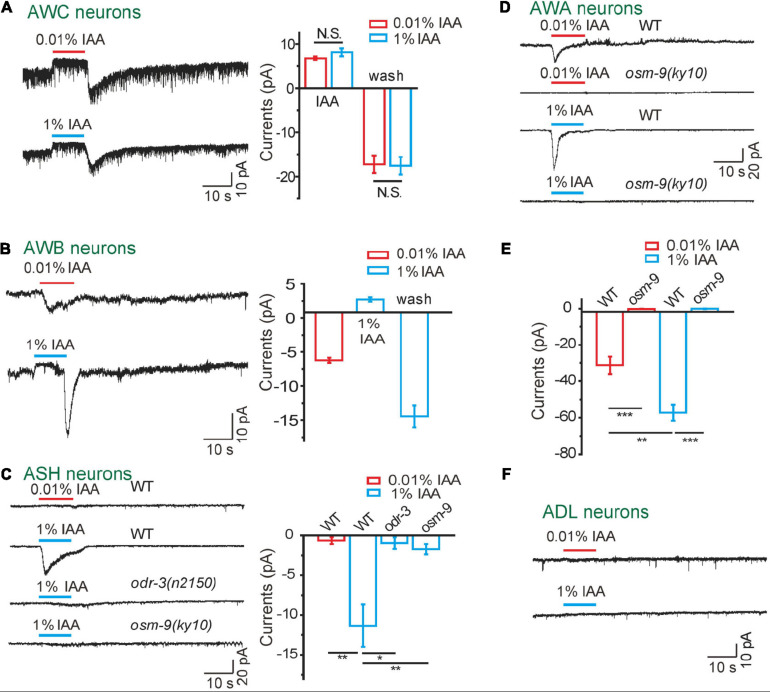
Odorants induced diverse currents from olfactory neurons in an intensity-dependent manner. **(A)** IAA induced bi-directional currents from AWC neurons. (Left) Representative traces. (Right) Quantification of the currents. *n* ≥ 6. **(B)** 1 and 0.01% IAA-induced opposite currents from AWB neurons. (Left) Representative traces. (Right) Quantification of the currents. *n* ≥ 6. **(C)** 1%, but not 0.01% IAA, induced inward currents from ASH neurons, which were mediated by ODR-3 and OSM-9. (Left) Representative traces. (Right) Quantification of the currents. *n* ≥ 7. **(D,E)** Both 0.01 and 1% IAA induced OSM-9-dependent inward currents from AWA neurons. **(D)** Representative traces. **(E)** Quantification of the currents. *n* ≥ 5. **(F)** Neither 0.01% nor 1% IAA induced detectable currents from ADL neurons. Representative traces were shown. *n* ≥ 6. The cell membrane potential was voltage-clamped at –70 mV. Error bars: SEM. N.S., not significant, *P < 0.05, **P < 0.01, ***P < 0.001.

AWC neurons are the main olfactory neurons mediating the attractive response to at least five attractive odorants including IAA, butanone, 2,3-pentanedione, benzaldehyde, and 2,4,5-trimethylthiazole (Bargmann, 2006). The pair of AWC is divided as the AWC^*ON*^ and another AWC^*OFF*^ based on the asymmetric expression pattern of the G protein-coupled serpentine receptor STR-2 (Bargmann, 2006; Chalasani et al., 2007). We tested the responses of AWC^*ON*^ and AWC^*OFF*^ to butanone, 2, 3-pentanedione, benzaldehyde, and IAA using *in vivo* patch clamp recording (Supplementary Figure 2A). Very interestingly, we found that butanone (0.01%) activated a sustained outward current followed by a large transient inward current upon washing off specifically in AWC^*ON*^ but not in AWC^*OFF*^, and 2,3-pentanedione (0.01%) triggered similar responses in AWC^*OFF*^ but not AWC^*ON*^ (Supplementary Figure 2B). Similarly, benzaldehyde (0.01%) and IAA (0.01%) activated bi-directional currents in both AWC^*ON*^ and AWC^*OFF*^ (Figure 1A and Supplementary Figure 2B). Our electrophysiological results of odorant-induced bi-directional currents are consistent with previously reported calcium imaging results that intracellular Ca^2+^ in AWC is decreased on odorant addition and increased upon odorant removal (Chalasani et al., 2007; Tsunozaki et al., 2008; Yoshida et al., 2012; Duan et al., 2020). The latency of IAA addition-induced outward currents in AWC was 195.7 ± 30.3 ms (*n* = 12), and that of IAA removal-induced inward currents was 1.25 ± 0.35 s (*n* = 12) (Supplementary Table 1), which are much slower than that of touch- receptor currents in touch receptor neurons in *C. elegans* (Kang et al., 2010; Geffeney et al., 2011; Li et al., 2011), supporting the previous reports that the intracellular second messenger pathways are involved in olfactory transduction.

A previous study has reported that AWC is suppressed by other neurons under high concentrations of IAA (Yoshida et al., 2012). We then asked whether high concentrations of IAA induce bi-directional currents in AWC neurons. Interestingly, we found that AWC responded to 0.01 and 1% IAA to a similar extend (Figure 1A). We thus tested the IAA (1%)-induced currents in *unc-13(e51)*, *unc-13(e1091)*, *unc-31(e928)*, and *unc-18(e81)* mutants, in which the release of neurotransmitter and/or neuropeptide is blocked (Hu et al., 2013; Zhou et al., 2013; Yang et al., 2015). Remarkably, AWC responded normally to 1% IAA in all these mutants (Supplementary Figure 2C). These results indicate that IAA-induced bi-directional currents in AWC are somehow concentration-independent, although low concentrations of IAA are attractive and high concentrations of IAA are repulsive to worms.

### Different Concentrations of Isoamyl Alcohol Induce Opposite Currents in Amphid Wing “B” Cell Neurons

We next checked IAA-induced currents in AWB neurons. High concentration of IAA (1%) induced outward currents in AWB and inward currents upon removal, which were similar to those in AWC (Figure 1B). Strikingly, 0.01% IAA induced inward instead of outward currents in AWB (Figure 1B). Moreover, AWB responded normally to both 0.01 and 1% IAA in *unc-13(e51)* mutants (Supplementary Figure 2D). Additionally, 0.01% IAA induced calcium responses in either *unc-13(e51)* or *unc-31(e928)* mutants were equal to that in wild-type worms (Supplementary Figures 2E,F). Taken together, these observations suggest that different concentrations of IAA may cell-autonomously induce opposite responses in AWB.

### Isoamyl Alcohol Induces TRPV/OSM-9-dependent Inward Currents in Amphid Neuron “H” Cell With Single Ciliated Sensory Ending and Amphid Wing “A” Cell (AWA) Neurons With Different Thresholds

By calcium imaging, it has been previously reported that polymodal nociceptor neuron ASH also responds to IAA (Yoshida et al., 2012; Duan et al., 2020). We found that only higher concentration of IAA (1%) activated inward currents in ASH (Figure 1C), which is consistent with previous reports that 1% IAA, but not 0.01% IAA, can trigger calcium elevations in ASH (Yoshida et al., 2012; Duan et al., 2020). Notably, the latency of IAA-induced currents in ASH was 0. 49 ± 0.14 s, and the rise time was 2 ± 0.3 s, both of which were slower than that of IAA-induced outward currents in AWC (Supplementary Table 1). Gα protein ODR-3 and TRPV channel OSM-9 are required for osmotic and repellent avoidance (Colbert et al., 1997; Kato et al., 2014; Duan et al., 2020). Consistently, IAA (1%) failed to induce any current in ASH neuron of either *odr-3(n2150)* or *osm-9(ky10)* mutants (Figure 1C), supporting that ASH relies on the ODR-3-OSM-9 pathway to detect high concentrations of IAA.

The olfactory neuron AWA shares some odorants sensation with AWC, such as 2,4,5-trimethylthiazole and IAA (Bargmann, 2006; Taniguchi et al., 2014). We found that both 1 and 0.01% IAA activated inward currents in AWA neurons in a dose-dependent manner (Figures 1D,E). Furthermore, no IAA-induced current was detected in AWA neurons of *osm-9(ky10)* mutants, suggesting that OSM-9 is required for the IAA-induced olfactory responses in AWA neurons (Figures 1D,E).

It has been implicated that ADL neurons are also involved in odorant sensing (Troemel et al., 1995). However, there was no detectable current recorded in ADL neurons in response to either high or low concentrations of IAA, suggesting that ADL neurons may not directly respond to IAA (Figure 1F).

### CNG Channel Mediates Isoamyl Alcohol-Induced Responses in Amphid Wing “C” Cell and Amphid Wing “B” Cell Neurons

**FIGURE 2:**
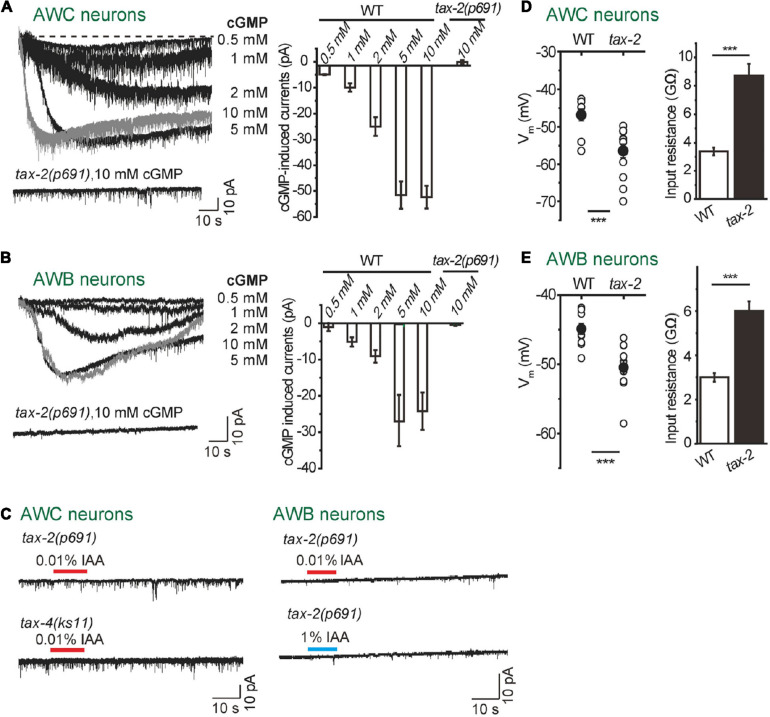
TAX-2/TAX-4 channel is critical for intensity-dependent olfactory encoding. **(A,B)** Dialyzing cGMP into AWC and AWB neurons with the recording pipette induced inward currents in wild-type worms, but not in *tax-2(p691)* mutants. **(A)** AWC neurons. **(B)** AWB neurons. The cell membrane potential was voltage-clamped at –70 mV. *n* ≥ 5. **(C)** No IAA-induced current was recorded in either *tax-2(p691)* or *tax-4(ks11)* mutants from AWC and AWB neurons. (Right) AWC neurons. (Left) AWB neurons. The cell membrane potential was voltage-clamped at –70 mV. *n* ≥ 6. **(D,E)** Membrane potentials of AWC neurons **(D)** and AWB neurons **(E)** were hyperpolarized and input resistances were increased in *tax-2(p691)* mutants. –10 mV voltage step was injected into AWC and AWB and the input resistance was calculated by –10 mV divided by the recorded currents. *n* ≥ 7. Error bars: SEM. ***P < 0.001.

A cyclic nucleotide-gated ion channel (CNG) encoded by two subunits of tax-2 (α-subunit) and tax-4 (β-subunit) is required for olfaction, thermosensation, phototransduction, gustation and O_2_-sensing in *C. elegans* (Coburn and Bargmann, 1996; Komatsu et al., 1996, 1999; Ward et al., 2008; Couto et al., 2013). By dialyzing cGMP into either AWC or AWB neurons with the recording pipette, we detected robust inward currents in a dose-dependent manner (Figures 2A,B). In *tax-2(p691)* mutant worms, the cGMP-induced currents were abolished in both AWC and AWB neurons (Figures 2A,B). Furthermore, in *tax-2(p691)* or *tax-4(ks11)* mutant background, IAA failed to induce any detectable current in either AWC or AWB neurons (Figure 1C). We also observed that the resting membrane potentials of both AWC and AWB were lower in *tax-2(p691)* mutants as compared to wild-type worms and the input resistances were higher (Figures 1D,E), indicating that some TAX-2/TAX-4 channels are constitutively open in these neurons.

Additional CNG channel homologs CNG-1 and CNG-3 are expressed by several amphid neurons including AWC (Cho et al., 2004, 2005; Smith et al., 2013). However, IAA-induced currents from AWC in *cng-1 (jh111)*;*cng-3 (jh113)* double mutants were similar to that of wild-type animals, suggesting that CNG-1 and CNG-3 are not required for olfactory transduction in AWC (Data not shown).

### Gα Proteins Are Differentially Activated by Different Concentrations of Isoamyl Alcohol

Heterotrimeric G proteins, composed of Gα, Gβ, and Gγ subunits, transduce signals from the plasma membrane receptors to intracellular effectors (Buck and Axel, 1991; Troemel et al., 1995; Jansen et al., 1999). *C. elegans* genome encodes 21 Gα, 2 Gβ, and 2 Gγ subunits. The Gi/o-like G_α_ protein ODR-3, GPA-2, GPA-3, GPA-5, and GPA-13 have been implicated in mediating olfactory-related behaviors in *C. elegans* (Roayaie et al., 1998; Hilliard et al., 2004; Lans et al., 2004; Taniguchi et al., 2014; Duan et al., 2020). We then investigated the role of G_α_ proteins in intensity-dependent IAA encoding.

**FIGURE 3:**
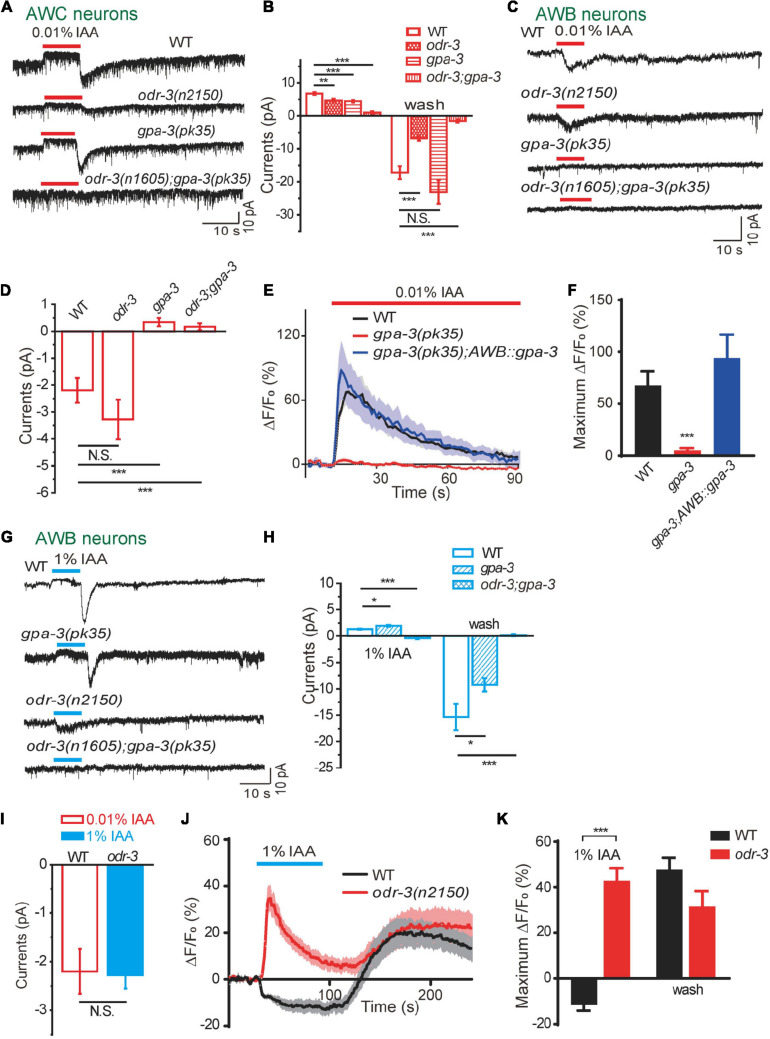
Two Gα proteins were differentially activated by different concentrations of IAA. **(A,B)** 0.01% IAA-induced currents from AWC neurons were mainly mediated by ODR-3. **(A)** Representative traces. **(B)** Quantification of the currents. *n* ≥ 6. **(C,D)** 0.01% IAA-induced inward currents from AWB neurons were mediated by GPA-3. **(C)** Representative traces. **(D)** Quantification of the currents. *n* ≥ 6. **(E,F)** 0.01% IAA-induced calcium increases from AWB neurons were mediated by GPA-3. **(E)** Representative traces. **(F)** Quantification of the maximumΔF/F_0_. *n* ≥ 7. **(G–I)** 1% IAA-induced outward currents from AWB neurons were mediated by ODR-3. **(G)** Representative traces. **(H,I)** Quantification of the currents. *n* ≥ 5. **(J,K)** 1% IAA-induced calcium decreases from AWB neurons were mediated by ODR-3. **(J)** Representative traces. **(K)** Quantification of the maximumΔF/F_0_. *n* ≥ 8. For patch-clamp recording, the cell membrane potential was voltage-clamped at –70 mV. Error bars: SEM. N.S., not significant, *P < 0.05, **P < 0.01, ***P < 0.001.

In AWC neurons, we found that both outward and inward currents were greatly reduced in *odr-3(n2150)* mutants, and the residual currents were eliminated in *odr-3(n1605);gpa-3(pk35)* double mutants. In *gpa-3(pk35)* mutants, the outward currents were slightly reduced, but the inward currents were unaffected (Figures 3A,B). These data suggest that ODR-3 mediates the majority of IAA-induced olfactory currents in AWC neurons.

In AWB neurons, both 1% and 0.01% IAA-induced currents were abolished in *odr-3(n1605);gpa-3(pk35)* double mutants, suggesting that ODR-3 and GPA-3 are also required for the IAA-mediated olfactory responses in AWB (Figures 3C,D,G,H). Nevertheless, low concentration (0.01%) of IAA-induced inward currents were abolished in *gpa-3(pk35)* mutants but kept normal in *odr-3(n2150)* mutants (Figures 3C,D), implying that GPA-3 alone was sufficient to mediate the low concentrations of IAA-induced olfactory responses in AWB. Consistently, 0.01% IAA-induced calcium responses were eliminated in *gpa-3(pk35)* mutants, and the deficit was fully rescued by expressing of ODR-3 specifically in AWB neurons (Figures 3E,F).

Very interestingly, 1% IAA induced inward currents in *odr-3(n2150)* mutants instead of the outward currents in the wild-type worms (Figures 3G–I). In fact, the inward currents induced by 1% IAA in *odr-3(n2150)* mutants were similar to those induced by 0.01% IAA in wild-type worms (Figure 3I). Notably, while intracellular Ca^2+^ in AWB neurons was decreased on 1% IAA addition and increased upon removal, 1% IAA induced robust calcium increases in AWB (Figures 3J,K). These data suggest that GPA-3 mediates low concentration of IAA-induced currents in AWB neurons, while ODR-3 is required for high concentration of IAA-induced responses in AWB.

### Guanylate Cyclases Are Essential for Intensity-Dependent Isoamyl Alcohol Encoding

How do ODR-3/GPA-3 regulate the open/closure of the CNG channel TAX-2/TAX-4? The TAX-2/TAX-4 channel is gated by intracellular cGMP (Komatsu et al., 1999; Ward et al., 2008; Wang et al., 2013). As the concentration of intracellular cGMP is determined by two enzymes, guanylate cyclase (GC, cGMP synthesis) and phosphodiesterase (PDE, cGMP hydrolysis) (Xiong et al., 1998; Yau and Hardie, 2009; Wang et al., 2013). We first tested how GCs are involved in the olfactory transduction of AWC and AWB neurons.

**FIGURE 4:**
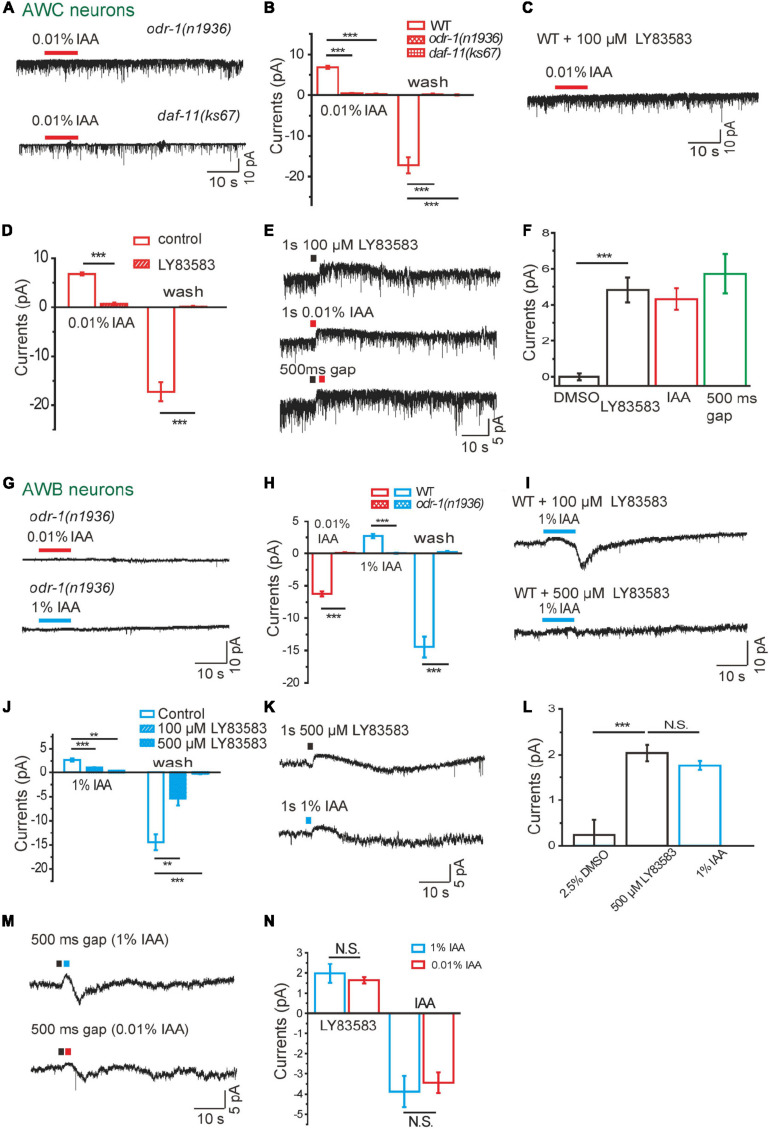
Guanylate cyclases are required in intensity-dependent olfactory encoding. **(A,B)** 0.01% IAA-induced currents from AWC neurons were abolished in either *odr-1(n1936)* or *daf-11(ks67)* mutant background. **(A)** Representative traces. **(B)** Quantification of the currents. *n* ≥ 6. **(C,D)** 0.01% IAA-induced currents from AWC neurons were blocked by 100 μM LY83583, a GC inhibitor, in the bath solution. **(C)** Representative traces. **(D)** Quantification of the currents. *n* ≥ 5. **(E,F)** A puff of either 100 μM LY83583 or 0.01% IAA induced an outward current from AWC neurons. **(E)** Representative traces. Black square: LY83583. Red square: 0.01% IAA. No summation of the currents induced by LY83853 and 0.01% IAA was observed, when a puff of LY83583 was followed by a successive puff of IAA within 500ms interval. **(F)** Quantification of the currents. A puff of 0.5% DMSO was taken as negative control. *n* ≥ 4. **(G,H)** Neither 0.01% nor 1% IAA elicited detectable current from AWB neurons in *odr-1(n1936)* mutants. **(G)** Representative traces. **(H)** Quantification of the currents. *n* ≥ 7. **(I,J)** 1% IAA-induced currents from AWB neurons were blocked by LY83583. **(I)** Representative trace. **(J)** Quantification of the currents. *n* ≥ 3. **(K,L)** A puff of either 500 μM LY83583 or 1% IAA induced an outward current from AWB neurons. **(K)** Representative traces. **(L)** Quantification of the currents. A puff of 2.5% DMSO was taken as negative control. *n* ≥ 3. **(M,N)** Transient LY83583-induced outward currents from AWB neurons were reversed to inward currents, when a successive puff of either 1 or 0.01% IAA was applied 500 ms after a puff of 500 μM LY83583. Black square: LY83583. Blue square: 1% IAA. Red square: 0.01% IAA. **(M)** Representative traces. **(N)** Quantification of the currents. The amplitudes of successive inward currents induced by 1 and 0.01% IAA were equal. *n* = 5. The cell membrane potential was voltage-clamped at –70 mV. Error bars: SEM. N.S., not significant, **P < 0.01, ***P < 0.001.

It have been reported that membrane guanylate cyclases ODR-1 and DAF-11 are involved in chemotaxis (Vowels and Thomas, 1994; L’Etoile and Bargmann, 2000). In AWC neurons, no IAA-induced current was recorded in either *odr-1(n1936)* or *daf-11(ks67)* mutants, suggesting that cGMP synthesis by ODR-1 and DAF-11 is essential for the olfactory transduction in AWC (Figures 4A,B). Consistently, IAA-induced currents were completely blocked by LY83583 (100 μM), a GC inhibitor (Su et al., 2006; Liu et al., 2010), in the bath solution (Figures 4C,D). We next used a previously described protocol to dissect the role of GCs in IAA-sensing (Su et al., 2006). We found that a 1 s puff of 100 μM LY83583 was sufficient to induce an outward current in AWC, similar in amplitude and the kinetics to that induced by a 1 s puff of 0.01% IAA (Figures 4E,F). To further test whether IAA reduced cGMP in AWC by inhibiting GCs, we applied a puff of LY83583 and a successive puff of IAA within 500 ms interval. We did not observe any summation effect between LY83583 and IAA application (Figures 4E,F). These results suggest that hyperpolarization of AWC with IAA addition mainly results from inhibition of GCs ODR-1/DAF-11, which decreases the intracellular level of cGMP, thus closes the CNG channel TAX-2/TAX-4.

In AWB neurons, we observed that both 1% and 0.01% IAA-induced currents were abolished in *odr-1(n1936)* mutants (Figures 4G,H). Similar to AWC neurons, application of GC inhibitor LY83583 in bath solution abolished 1% IAA-induced responses in AWB (Figures 4I,J). A saturating puff (500 μM, 1 s) of LY83583 induced an outward current similar to that induced by a puff (1 s) of 1% IAA (Figures 4K,L). Interestingly, with a puff of either 0.01 or 1% IAA after a saturating puff of LY83583 in rapid succession, LY83583-induced outward current was reversed to inward current by IAA (Figures 4M,N). These results suggest that 1% IAA-induced outward currents from AWB, but not 0.01% IAA-induced inward currents, results from inhibition of membrane GCs.

### Phosphodiesterases Play a Modulatory Role in Isoamyl Alcohol-Sensing of Amphid Wing “C” Cell Neurons

**FIGURE 5:**
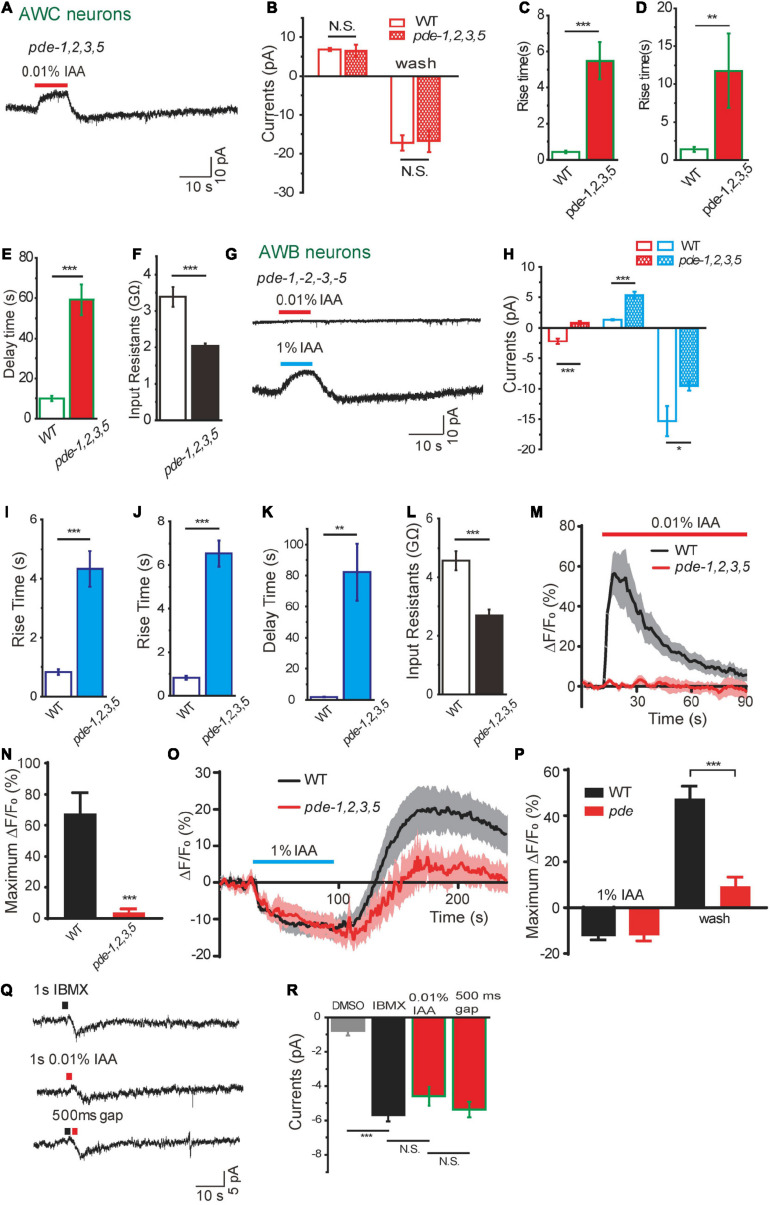
Dual functions of phosphodiesterases in intensity-dependent olfactory encoding. **(A–F)** 0.01% IAA-induced currents from AWC neurons in *pde–1(nj57);–2(nj58);–3(nj59);–5(nj49)* quadruple mutants. **(A)** Representative trace. **(B)** The amplitude of olfactory currents was normal in *pde* quadruple mutants. The rise time of both outward currents **(C)** and inward currents **(D)** become slower, and the decay time of inward currents induced by odorant removal become much longer **(E)**. **(F)** Input resistance of AWC neurons was decreased in *pde* quadruple mutants. *n* ≥ 6. **(G–L)** 0.01 and 1% IAA-induced currents from AWB neurons in *pde–1(nj57);–2(nj58);–3(nj59);–5(nj49)* quadruple mutants. **(G)** Representative traces. **(H)** Quantification of the currents. 0.01% IAA-induced currents from AWB neurons were abolished in *pde* quadruple mutants. The amplitudes of 1% IAA-induced outward currents were increased and inward currents were decreased in *pde* quadruple mutants. The rise times of both 1% IAA-induced outward currents **(I)** and inward currents **(J)** become much slower, and the decay time of inward currents induced by 1% IAA removal become much longer **(K)**. Input resistance of AWB neurons was decreased in *pde* quadruple mutants **(L)**. *n* ≥ 6. **(M,N)** 0.01% IAA-induced calcium increases in AWB neurons were abolished in *pde* quadruple mutants. **(M)** Representative traces. **(N)** Quantification of the maximumΔF/F_0_. *n* ≥ 12. **(O,P)** 1% IAA-induced calcium decreases in AWB neurons were retained in *pde* quadruple mutants. **(O)** Representative traces. **(P)** Quantification of the maximumΔF/F_0_. *n* ≥ 6. **(Q,R)** A puff of either 1 mM IBMX or 0.01% IAA induced an inward current from AWB neurons. **(Q)** Representative traces. **(R)** Quantification of the currents. Black square: IBMX. Red square: 0.01% IAA. A puff of 0.5% DMSO was taken as negative control. No summation of the currents induced by IBMX and 0.01% IAA was observed, when a puff of IBMX was followed by a successive puff of 0.01% IAA within 500ms interval. *n* ≥ 3. Error bars: SEM. N.S., not significant, *P < 0.05, **P < 0.01, ***P < 0.001.

Decrease of cGMP can be achieved by more degradation, less synthesis, or both. There are six PDEs in *C. elegans*, in which PDE-2 and PDE-4 cleave cAMP, while PDE-1, PDE-2, PDE-3, and PDE-5 can cleave cGMP (Liu et al., 2010; Couto et al., 2013; Wang et al., 2013). PDEs have been reported to be essential for olfactory adaptation in *C. elegans* (O’Halloran et al., 2012). However, it is unclear whether PDEs are involved in acute odorant sensing in *C. elegans*. We found the amplitudes of IAA-induced olfactory currents from AWC from *pde-1(nj57);pde-2(nj58);pde-3(nj59);pde-5(nj49)* quadruple mutants were not significantly different from those in wild-type worms (Figures 5A,B). However, the kinetics of the currents were dramatically changed (Figures 5A,C–E). Loss of PDEs reduced rise rates of both the outward and inward currents. Recovery rate of the inward current was also prolonged in *pde* quadruple mutant animals (Figures 5A,C–E). These observations suggest that PDEs play a modulatory role in olfactory transduction of AWC. We found that the input resistance of AWC in *pde* quadruple mutants was smaller than that in wild-type worms, indicating that more CNG channels were open in AWC of *pde* quadruple mutants, probably due to a higher level of intracellular cGMP (Figure 5F). In addition, IAA-induced currents in AWC neurons showed no difference between WT and *pde-4(nj60);pde-6(ok3410)* double mutants, suggesting that cAMP is not required for IAA-sensing in AWC neurons (Data not shown).

### Dual Roles of Phosphodiesterase in Intensity-Dependent Isoamyl Alcohol Encoding in Amphid Wing “B” Cell Neurons

We then sought to characterize the function of PDEs in intensity-dependent IAA encoding in AWB neurons. In *pde-1(nj57);pde-2(nj58); pde-3(nj59); pde-5(nj49)* quadruple mutants, the amplitude of 1% IAA addition-induced outward currents was increased and that of 1% IAA removal-induced inward currents was reduced (Figures 5G,H). Similar to what we observed from AWC, the kinetics of both outward and inward currents induced by 1% IAA from AWB were dramatically changed (Figures 5G,I–K). Loss of PDEs also reduced the input resistance of AWB, indicating that the level of cGMP in AWB neurons was lower and less CNG channels were open in *pde* quadruple mutant animals (Figure 5L). These results indicate that PDEs also play a modulatory role in 1% IAA-induced responses in AWB neurons.

Surprisingly, 0.01% IAA-induced currents from AWB were absent in *pde* quadruple mutants (Figures 5G,H). Consistently, 0.01% IAA-induced calcium increases in AWB neurons were abolished, while 1% IAA-induced calcium decreases were retained in *pde* quadruple mutants (Figures 5M–P). We speculated that 0.01% IAA-induced inward currents resulted from inhibition of PDEs, thereby increasing the level of cGMP and opening of more CNG channels. To further test this hypothesis, we used a PDE inhibitor 3-isobutyl-1-methyl-xanthine (IBMX) (Su et al., 2006; Liu et al., 2010). A puff of IBMX (1 mM, 1 s) induced an inward current identical to that induced by a puff of 0.01% IAA (Figures 5Q,R). When we applied a puff of IBMX and a successive puff of 0.01% IAA within 500 ms interval, IAA elicited an inward current only when the IBMX-induced current declined from the peak (Data now shown). There was no summation effect between IBMX and 0.01% IAA application, although the IBMX-induced current in AWB neurons (–5.7 ± 0.4 pA) was only a small fraction of saturated cGMP-induced current (–26.8 ± 7.1 pA, 5 mM cGMP) (Figures 2B, 5Q,R). These results support the notion that 0.01% IAA induces inward currents from AWB neurons by inhibiting PDEs.

### Phosphodiesterase-5 Is Involved in Isoamyl Alcohol-Sensing in Amphid Wing “B” Cell Neurons

**FIGURE 6:**
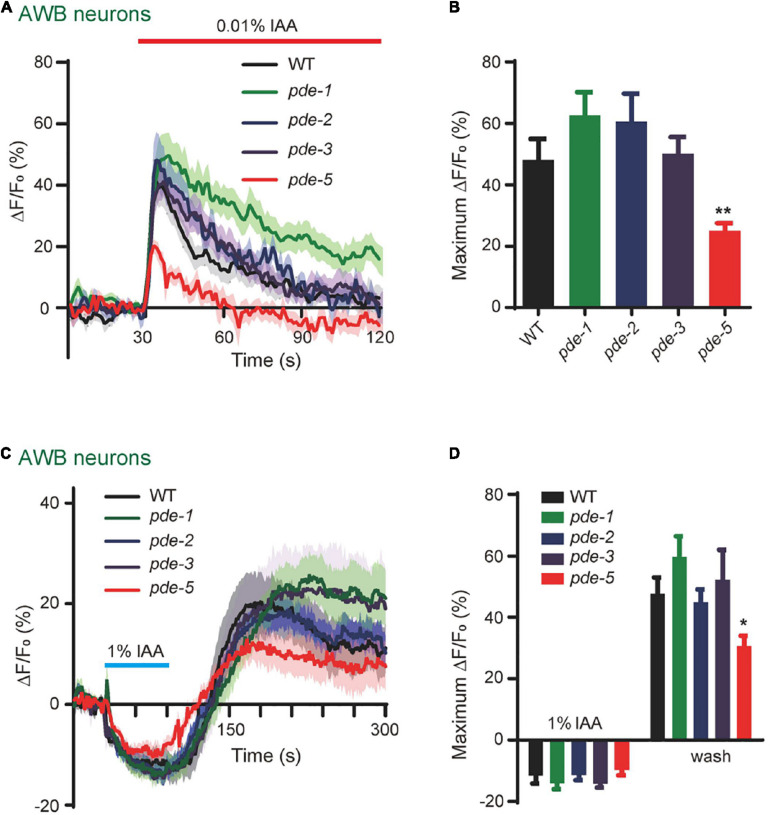
PDE-5 is involved in olfactory encoding in AWB neurons. **(A,B)** 0.01% IAA-induced calcium increases in AWB neurons were reduced in *pde-5(nj49)* mutants. **(A)** Representative traces. **(B)** Quantification of the maximumΔF/F_0_. *n* ≥ 10. **(C,D)** 1% IAA removal-induced calcium increases in AWB neurons were reduced in *pde-5(nj49)* mutants. **(C)** Representative traces. **(D)** Quantification of the maximumΔF/F_0_. *n* ≥ 11. Error bars: SEM. N.S., not significant, *P < 0.05, **P < 0.01.

Considering that PDEs play differential roles in response to different concentrations of IAA in AWB neurons (Figure 5), we next tested whether different subtypes of PDE are differentially involved in these events. We found that 0.01% IAA elicited-calcium increases were significantly reduced in *pde-5(nj49)* mutant animals, but not in either *pde-1(nj57) or pde-2(nj58) or pde-3(nj59)* mutants, suggesting that PDE-5 plays a role in 0.01% IAA-induced responses in AWB neurons (Figures 6A,B). Notably, the calcium increases elicited by removal of 1% IAA were also reduced in *pde-5(nj49)* mutants (Figures 6C,D). Given that calcium increases elicited by either high or low concentrations of IAA were not absent from any *pde* single mutant, several PDE proteins may function redundantly in AWB neurons.

## Discussion

In this study, we demonstrate that a set of olfactory neurons combinatorically employ multiple signaling pathways to detect a single odor in an intensity-dependent manner. Our data showed that AWC neurons detect IAA through a Ga protein-cGMP-CNG channel pathway. Meanwhile, AWB neurons differentially respond to high and low concentrations of IAA, mediated by two antagonistic Ga protein-cGMP-CNG channel signaling pathways. Together with the notion that AWA and ASH neurons respond to IAA via Ga protein-OSM-9 channel pathway with different thresholds (Figure 1), our observations reveal how *C. elegans* utilizes a complex combination of olfactory sensory neurons and intracellular signaling pathways to detect and discriminate intensity of olfactory cues.

A combinatorial receptor coding scheme is often employed to encode odorant identities in the animal kingdom (Malnic et al., 1999; DeMaria and Ngai, 2010). A single odorant can activate multiple types of odorant receptors and a single receptor can also respond to multiple odorants. The *C. elegans* olfactory system operates according to the one neuron–multiple receptors mode (Bargmann et al., 1993), which is different from the one neuron–one receptor mode in mammals (Malnic et al., 1999; DeMaria and Ngai, 2010). However, a similar coding scheme is likely employed in *C. elegans*. For example, two GPCRs SRI-14 and ODR-10 were reported to serve as receptors of different concentrations of diacetyl in AWA neurons (Taniguchi et al., 2014), while our recently published study identified STR-61 and SRH-79 as IAA receptors with different affinities in ASH neurons and the amphid sheath glia, respectively (Duan et al., 2020). Logically, two types of IAA receptors may be expressed in AWB neurons. One is a low affinity receptor with high threshold, which can only detect high concentration of IAA and is associated with Gα protein ODR-3. Another is a high affinity receptor with low threshold, which is more sensitive to IAA and is associated with Gα protein GPA-3. However, in AWC neurons, a high affinity IAA receptor may be expressed and is associated with ODR-3. In addition, high affinity IAA receptor and low affinity IAA receptor are expressed in AWA and ASH neurons, respectively, which are associated with ODR-3. It should be noted that the IAA receptors expressed in AWB, AWC, ASH, and AWA neurons may differ, and it would be very interesting to identify these receptors in further studies.

**FIGURE 7:**
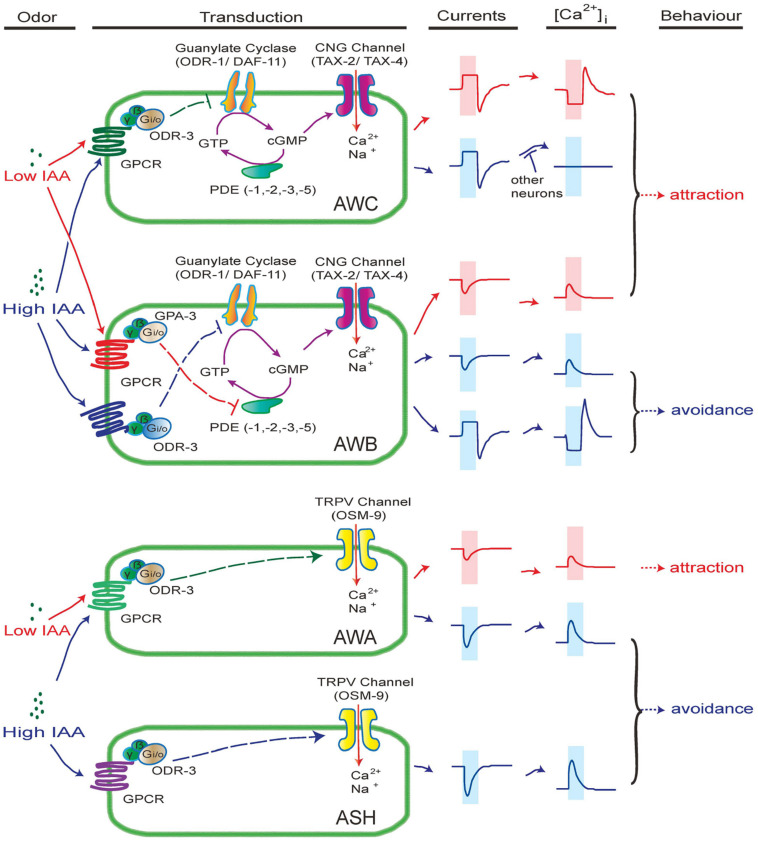
A working model of intensity-dependent olfactory encoding in *C. elegans*. In this model, different receptors (GPCRs) with different affinities are expressed in olfactory neurons. Gα proteins ODR-3 and GPA-3 are differentially associated with the receptors. The CNG channel TAX-2/TAX-4 is constantly open in resting state in AWB and AWC neurons. In AWC neurons, binding of IAA to its receptor mainly activates Gα ODR-3, thereby inhibiting the activity of membrane GCs ODR-1/DAF-11. Consequentially, cGMP synthesis is reduced, thereby closing cGMP-gated CNG channel TAX-2/TAX-4 and hyperpolarizing AWC. PDEs set the basal level of cGMP in AWC and modulate the kinetics of IAA-induced response. Removal of IAA relieves the inhibition of GCs and leads to depolarization of AWC. In AWB neurons, low concentrations of IAA may bind to a high affinity IAA receptor, which is associated with Gα GPA-3, thereby inhibiting PDEs and increasing cGMP. Thus, more CNG channels open and AWB neurons get depolarized. High concentrations of IAA may bind to both high and low affinity IAA receptors. When Gα ODR-3-associated low affinity receptor is activated, the activity of membrane GCs are inhibited, thereby hyperpolarizing AWB via a pathway similar to that in AWC. In addition, a high affinity IAA receptor and a low affinity IAA receptor are likely expressed in AWA and ASH neurons, respectively, which are associated with ODR-3. Activation of ODR-3 leads to closure of TRPV channel OSM-9, thereby depolarizing and activating AWA and ASH neurons. Note that the IAA receptors expressed in AWB, AWC, ASH and AWA neurons may be different.

Even though many similarities stand between vertebrates and *C. elegans* olfactory receptor neurons, the olfactory signaling cascades in *C. elegans* are diverse and show more similarities with phototransduction in some vertebrates and invertebrates. Olfactory transduction in AWC and AWB neurons are mediated by cGMP signaling pathways (Figure 2), which are similar to phototransduction in ciliary photoreceptors in vertebrate rods and cones, lizard parietal eye, and *C. elegans* ASJ neurons (Su et al., 2006; Yau and Hardie, 2009; Liu et al., 2010). The balance of cGMP relies on the homeostasis between synthesis (GC) and hydrolysis (PDE) (Yau and Hardie, 2009). Our electrophysiological data suggest that CNG channel TAX-2/TAX-4 is constantly open in the resting state in AWC and AWB neurons (Figure 2). An appealing model is as following (Figure 7): in AWC neurons, odorants bind to their receptor GPCR and activate Gα proteins ODR-3, which in turn inhibits membrane GCs ODR-1/DAF-11 and leads to drop of the intracellular level of cGMP, thereby closing CNG channels TAX-2/TAX-4 and hyperpolarizing the cell. Guanylate cyclase, but not phosphodiesterase, is likely a key component in this event. Firstly, the GC inhibitor LY83583 induced the same outward current as IAA (Figures 4E,F). Secondly, the kinetics, but not the amplitude of IAA-induced currents from AWC were significantly changed in *pde-1(nj57);pde-2(nj58);pde-3(nj59);pde-5(nj49)* quadruple mutants (Figures 5A,C–E), indicating that PDEs are not required for the olfactory transduction, but instead play a modulatory role in the olfactory transduction in AWC neurons. The G_*o/i*_-GC cGMP signaling pathway in AWC neurons is likely different from which in vertebrate photoreceptor rods and cones and in the parietal eye photoreceptor cells. In vertebrate photoreceptor rods, G_*t*_/G_*gust*_ activates PDE and leads to the hydrolysis of cGMP, thereby closing the CNG channels that are open in darkness and finally hyperpolarizing the cells (Xiong et al., 1998; Yau and Hardie, 2009). In the vertebrate parietal eye photoreceptor cells, two antagonistic light signaling pathways present in the same cell- a blue-sensitive hyperpolarizing pathway and a green-sensitive depolarizing pathway (Su et al., 2006). The hyperpolarizing pathway is mediated by activate a PDE by pinopsin- G_*gust*_, and the depolarizing pathway is mediated by inhibit a PDE by parietopsin- G_*o*_ (Su et al., 2006). Nevertheless, G protein-GC pathway is present in scallop hyperpolarizing photoreceptors, in which G_*o*_ activates a GC and increases intracellular cGMP, thereby opening a cGMP-gated K channel and hyperpolarizing the cell (Gomez and Nasi, 2000; Yau and Hardie, 2009). Notably, G_*o*_ activates a GC in scallop hyperpolarizing photoreceptors, whereas G_*o/i*_ may inhibit GCs in AWC neurons (Figure 4). Thus, the cGMP signaling pathway in AWC seems to be unique in evolution. Our data show that PDEs have modulatory function in acute odorant sensing in AWC and AWB. Notably, all though PDEs have previously reported to be involved in phototransduction in ASJ neurons and thermosensory transduction in AFD neurons, their function in these neurons is likely different from that in AWC and AWB neurons. In ASJ neurons, UV light-induced inward currents became much larger and more sustained in PDEs mutants (Liu et al., 2010). In AFD neurons, loss of PDEs increased threshold temperature T^∗^ and induced longer-lived thermoreceptor currents (ThRCs), but did not change the amplitude of ThRCs (Wang et al., 2013).

In AWB neurons, high and low concentrations of IAA induced opposite currents, which is consistent to previously reported observations that AWB contributes to both avoidance response to high concentration of IAA and chemotaxis to low concentration of IAA (Yoshida et al., 2012). Here we revealed that high concentration of IAA (1% IAA) induced outward currents and calcium decreases from AWB neurons through a Gα (ODR-3)-membrane GCs (ODR-1/DAF-11)-cGMP pathway, which is similar to olfactory transduction in AWC neurons. However, low concentration of IAA (0.01%) induced inward currents and calcium increases from AWB neurons through a Gα(GPA-3)-PDEs (PDE–1,–2,–3,–5) pathway, which is evolutionarily conserved to the depolarizing phototransduction in vertebrate parietal eye photoreceptor cells (Xiong et al., 1998; Yau and Hardie, 2009; Figure 7). Low concentration of IAA-induced inward currents from AWB are similar to those induced by UV light from ASJ. However, loss of PDEs abolished the currents from AWB, but increased those from ASJ (Liu et al., 2010), indicating that the underlying mechanisms may be different.

Taken together, our study demonstrated how multiple signal transduction pathways are combinatorically employed by a set of olfactory sensory neurons to detect and discriminate intensity of olfactory inputs. Interestingly, the phenomenon that different concentrations of IAA induce opposite currents from AWB neurons resembles antagonistic light sensing in vertebrate parietal-eye photoreceptor. Furthermore, low concentration of IAA induces currents from AWB neurons through a mechanism conserved to that in vertebrate parietal-eye photoreceptor. These observations suggest a potential evolutionary scheme of olfactory transduction and phototransduction.

## Materials and Methods

### Key Resources Table

**Table:** 

**Reagent or Resource**	**Source**	**Identifier**
**Chemicals, Peptides, and Recombinant Proteins**		
2, 3, 5-Trimethylthiazole	Sigma-Aldrich	Cat#W332518
Benzaldehyde	Sigma-Aldrich	Cat#B1334
Isoamyl alcohol	Sigma-Aldrich	Cat#W205702
Ethyl alcohol	Sigma-Aldrich	Cat#459836
2,3-pentanedione	Sigma-Aldrich	Cat#A5835
LY83583	Sigma-Aldrich	Cat#A6563
3-isobutyl-1-methyl-xanthine	Sigma-Aldrich	Cat#I5879
**Critical Commercial Assays**		
In-Fusion HD Cloning Kit	Takara	Cat#639649
**Experimental Models: Organisms/Strains**		
*N*_2_	CGC	ST348
*oyIs44[odr-1:RFP]*	CGC	PY2417
*tax-4(ks11);oyIs44[Podr-1:RFP]*	This study	ST426
*tax-2(P691),oyIs44[Podr-1:RFP]*	This study	ST481
*unc-13(e1091),oyIs44[Podr-1:RFP]*	This study	ST483
*unc-13(e51),oyIs44[Podr-1:RFP]*	This study	ST486
*kanEx020[Pstr-2:GFP];oyIs44[Podr-1:RFP]*	This study	ST523
*unc-31(e928);oyIs44[Podr-1:RFP]*	This study	ST576
*unc18(e81);oyIs44[Podr-1:RFP]*	This study	ST497
*N2;kanEx22[Pstr-2:GFP]*	This study	ST490
*pde-1(nj57);pde-2(nj58);pde-3(nj59);pde-5(nj49);kanEx22[Pstr-2:GFP]*	This study	ST600
*pde-4(nj60),pde-6(ok2410);kanEx22[Pstr-2:GFP]*	This study	ST595
*odr-3(n2150);kanEx22[Pstr-2:GFP]*	This study	ST601
*gpa-3(pk35); kanEx22 [Pstr-2:GFP]*	This study	ST909
*odr-1(n1936)*; *oyIs44[Podr-1:RFP]*	This study	ST540
*nt1/daf-11(m47);oyIs44[Podr-1:RFP]*	This study	ST541
*gpa-3(pk35); odr-3(n1605);kanEx22 [Pstr-2:GFP]*	This study	ST645
*kanEx41[Podr-1:DsRed2b]*	This study	ST656
*kanEx23[Psra-6:Dsred* + *Pstr-3:yfp]*	This study	ST717
*osm-9(ky10);kanEx23[Psra-6:Dsred* + *Pstr-3:yfp]*	This study	ST718
*odr-3(n2150);kanEx23[Psra-6:Dsred* + *Pstr-3:yfp]*	This study	ST550
*cng-1(jh111); cng-3(jh113);kanEx41[Podr-1:Dsred2b]*	This study	ST637
*odr-3(n2150); kanEx41[Podr-1:DsRed2b]*	This study	ST772
*kanEx81[Pstr-1:Dsred]*	This study	ST793
*pde-1(nj57);pde-2(nj58);pde-3(nj59);pde-5(nj49); kanEx81[Pstr-1:Dsred]*	This study	ST794
*odr-3(n2150); kanEx81[Pstr-1:Dsred]*	This study	ST834
*gpa-3(pk35);odr-3(n1605); kanEx81[Pstr-1:Dsred]*	This study	ST838
*gpa-3(pk35); kanEx81[Pstr-1:Dsred]*	This study	ST906
*kanEx266[Pstr-1:mcherry* + *Pstr-1:GCaMP5.0* + *Plin-44:gfp]*	This study	ST1890
*pde-1(nj57); kanEx266[Pstr-1:mcherry* + *Pstr-1:GCaMP5.0* + *Plin-44:gfp]*	This study	ST1740
*pde-2(nj58); kanEx266[Pstr-1:mcherry* + *Pstr-1:GCaMP5.0* + *Plin-44:gfp]*	This study	ST1736
*pde-3(nj59); kanEx266[Pstr-1:mcherry* + *Pstr-1:GCaMP5.0* + *Plin-44:gfp]*	This study	ST1738
*pde-5(nj49); kanEx266[Pstr-1:mcherry* + *Pstr-1:GCaMP5.0* + *Plin-44:gfp]*	This study	ST1742
*pde-1(nj57);pde-2(nj58);pde-3(nj59);pde-5(nj49); kanEx266[Pstr-1:mcherry* + *Pstr-1:GCaMP5.0* + *Plin-44:gfp]*	This study	ST1313
*odr-3(n2150); kanEx266[Pstr-1:mcherry* + *Pstr-1:GCaMP5.0* + *Plin-44:gfp]*	This study	ST1310
*gpa-3(pk35); kanEx266[Pstr-1:mcherry* + *Pstr-1:GCaMP5.0* + *Plin-44:gfp]*	This study	ST1311
*kanEx277[Psrh-220:mcherry* + *Plin-44:gfp]*	This study	ST1320
*unc-13(e51); kanEx266[Pstr-1:mcherry* + *Pstr-1:GCaMP5.0* + *Plin-44:gfp]*	This study	ST1427
*unc-31(e1091);kanEx266[Pstr-1:mcherry* + *Pstr-1:GCaMP5.0* + *Plin-44:gfp]*	This study	ST1428
*gpa-3(pk35);kanEx480[Pstr-1:gpa-3 cDNA:sl2a-tagRFP* + *Pstr-1:GCaMP5.0* + *Plin-44:gfp]*	This study	ST1700
*kanEx52[Podr-10:mCherry* + *Punc-122:gfp]*		This study	ST568
*osm-9(ky10);kanEx52[Podr-10:mCherry* + *Punc-122:gfp]*		This study	ST569
**Software and Algorithms**			
ImageJ	NIH	https://imagej.nih.gov/ij/; RRID:SCR_003070	
Micro-Manager	Vale Lab, UCSF	http://micro-manager.org, RRID:SCR_000415.	

### Contact for Reagent and Resource Sharing

Requests for information and for resources and reagents should be directed to and will be fulfilled by the Lead Contact, Lijun Kang (kanglijun@zju.edu.cn).

### Strains and Media

All nematode strains were cultivated at 20°C on nematode growth medium (NGM) plates seeded with the OP50 strain of *Escherichia coli* using standard methods previously described (Brenner, 1974). Well-fed Day 2 adult worms were used in all experiments unless otherwise indicated. The strains used are listed in the Key Resources Table. wild-type N2, *tax-2(p691)*, *tax-4(ks11)*, *cng-1(jh111);cng-3(jh113)*, *unc-13(e51)*, *unc-13(e1091)*, *unc-31(e928)*, *unc-18(e81)*, *odr-3(n2150)*, *gpa-3(pk35)*, *odr-3(n1605);gpa-3(pk35)*, *odr-1(n1936)*, *daf-11(ks67)*, *osm-9(ky10)*, *pde-1(nj57), pde-2(nj58), pde-3(nj59), pde-5(nj49), pde-1(nj57);pde-2(nj58);pde-3(nj59);pde-5(nj49)* and *pde-4(nj60);pde-6(ok3410)* were provided by the *Caenorhabditis* Genetic Center (CGC).

### Molecular Biology

Promoters were PCR-amplified from N2 genomic DNA and then recombined with specific donor vector fragments using the In-Fusion PCR Cloning Kit (TaKaRa Inc.). *Podr-1:RFP* (AWC and AWB), *Podr-1:DsRed2b* (AWC and AWB), *Pstr-2:DsRED* (AWC^*on*^), *Pstr-1:DsRED* (AWB), *Psra-6:DsRED* (ASH and ASI), *Pstr-3:YFP* (ASI), *Podr-10:mCherry* (AWA*)* and *Psrh-220:mCherry* (ADL) transgenic strains were used to identify AWC, AWB, ASH, AWA and ADL neurons, respectively. *Pstr-1:GCaMP5.0* was constructed for calcium imaging in AWB neurons.

### Chemotaxis Assays

Chemotaxis assays were performed essentially as described (Yoshida et al., 2012). Briefly, 1 μl of 1M sodium azide was spotted on both marks of the plate. One μl 1% diluted (in ethanol) odorant or 10 μl undiluted odorant was spotted on one mark of the plate and the same amount of ethanol was placed on the opposite mark. After 1 h, the number of the worms in the odor area and control area were calculated. Chemotaxis index = [(number of the worms in the odor area) − (number of the worms in the control area)]/[(number of the worms in the odor area) + (number of the worms in the control area)].

### Electrophysiology

Patch-clamp recording was carried out on an Olympus microscope (BX51WI) with an Axon 700A patch-clamp amplifier and Digidata 1320A interface (Axon Instruments). Signals were filtered at 2 kHz using amplifier circuitry, sampled at 10 kHz, and analyzed using Clampex 10.4 (Axon Instruments). Recording pipettes were pulled from borosilicate glass capillaries (B-120-69-10, Sutter Instruments) to a resistance of 15–20 MΩ on a P-97 micropipette puller (Sutter Instruments). Worms were glued on the surface of a sylgard-coated cover glass. A small piece of cuticle in the head of the worm was cut open and glued to the coverslip to expose the neurons of interest for recording as previously described (Yue et al., 2018; Zou et al., 2018). Odorants were delivered to the nose of the worm using a glass capillary with a tip I.D. of ∼110 μm and were washed off with bath solution using another glass capillary with a tip I.D. of ∼220 μm. The odorant applying and washing commands were driven by the Digidata 1320A interface. The bath solution constitutes (mM) of 145 NaCl, 2.5 KCl, 1 CaCl_2_, 1 MgCl_2_, 20 D-glucose and 10 HEPES (325∼335 mOsm, pH adjusted to 7.3). The pipette solution constitutes (mM) of 145 potassium gluconate, 2.5 KCl, 5 MgCl_2_, 10 HEPES, 0.25 CaCl_2_, 10 glucose, 5 EGTA, 5 Na_2_ATP and 0.5 NaGTP (315∼325 mOsm, pH adjusted to 7.2).

### Calcium Imaging

Animals were immersed in bath solution (145 mM NaCl, 2.5 mM KCl, 1 mM MgCl2, 5 mM CaCl2, 10 mM HEPES, 20 mM glucose, pH adjusted to 7.3 with NaOH) and subsequently glued on a glass coverslip with a medical grade cyanoacrylate-based glue (Gluture Topical Tissue Adhesive, Abbott Laboratories) (Yue et al., 2018; Duan et al., 2020). All odorants were first dissolved 1:1 in DMSO as stock solutions and then diluted in the bath solution prior to use. The green fluorescent GCaMP5.0 was used to measure the intracellular calcium signals (Yue et al., 2018; Duan et al., 2020). Fluorescent images were acquired using an Olympus microscope (IX71) under a 40x objective lens coupled with an Andor DL-604M EMCCD camera. Data were collected using the Micro-Manager software. GCaMP5.0 was excited by a ThorLabs blue light (460–480 nm) LED lamp. The fluorescent signals were collected with a 1 Hz sampling rate.

### Statistical Analysis

Data analysis was performed using GraphPad Prism 6. All data were presented as mean ± SEM. Unpaired two-tailed Student’s *t*-test or two-way ANOVA was used to compare data sets. If the data were not normally distributed, the Wilcoxon test was used. *P* < 0.05 was considered to be statistically significant. Sample sizes were determined by the reproducibility of the experiments and are similar to the sample sizes generally used in the field.

## Data Availability Statement

The original contributions presented in the study are included in the article/Supplementary Material, further inquiries can be directed to the corresponding author/s.

## Author Contributions

LK and SD designed the experiments. HC conducted the calcium imaging experiments. YL conducted the electrophysiological experiments. YX, JS, ZT, and SL conducted the other experiments. HC and YL contributed equally to this study. YL, HC, and LK analyzed and interpreted the results. YL and LK wrote the manuscript. All authors contributed to the article and approved the submitted version.

## Conflict of Interest

The authors declare that the research was conducted in the absence of any commercial or financial relationships that could be construed as a potential conflict of interest.

## Publisher’s Note

All claims expressed in this article are solely those of the authors and do not necessarily represent those of their affiliated organizations, or those of the publisher, the editors and the reviewers. Any product that may be evaluated in this article, or claim that may be made by its manufacturer, is not guaranteed or endorsed by the publisher.

## Supplementary Material

Supplementary Figure 1 Odorants which are attractive in low concentration elicit avoidance response with high concentration in *C. elegans*. Related to Figure 1. Odorants were diluted with ethanol. IAA, isoamyl alcohol; Tt, 2,4,5-trimethylthiazole; Bz, benzaldehyde; Pd, 2,3-pentanedione. *N* = 3. Error bars: SEM.

Supplementary Figure 2 IAA induced responses in AWC and AWB neurons. Related to Figure 1. (A) A schematic drawing of the calcium imaging and electrophysiological recording of odorant-induced responses in *C. elegans* olfactory neurons. **(B)** Representative traces show olfactory currents from AWC neurons induced by 0.01% Bu (AWC^*ON*^), 0.01% Bz (AWC^*ON*^ and AWC^*OFF*^), and 0.01% Pd (AWC^*OFF*^). Bz, benzaldehyde. Pd, 2,3-pentanedione. Bu, butanone. **(C)** 1% IAA-induced currents in AWC neurons in *unc-13(e51), unc-13(e1091), unc-31(e928)* and *unc-18(e81)* mutants were equal to that in wild-type animals. *n* ≥ 4. **(D)** IAA-induced currents in AWB neurons in *unc-13* mutants. *n* ≥ 7. **(E,F)** 0.01% IAA-induced calcium responses in AWB neurons in *unc-13(e51)* and *unc-31(e928)* mutants. *n* ≥ 7. For patch-clamp recording, the cell membrane potential was voltage-clamped at –70 mV. Error bars: SEM. N.S., not significant.

Supplementary Table 1 Biophysical properties of IAA-induced currents from AWC and ASH neurons. Related to Figure 1. The rise times and decay times were obtained from 10 to 90% of the peak amplitude. Voltage: –70 mV. *n* ≥ 5.
