# Supplementary material for: Molecular Strategies for Intensity-Dependent Olfactory Processing in Caenorhabditis elegans
Source: Front Mol Neurosci. 2021 Nov 4;14:748214. doi: 10.3389/fnmol.2021.748214 (PMC8600271; doi:10.3389/fnmol.2021.748214)
Supplement: Supplementary Figure 1 — Odorants which are attractive in low concentration elicit avoidance response with high concentration in C. elegans. Related to Figure 1. Odorants were diluted with ethanol. IAA, isoamyl alcohol; Tt, 2,4,5-trimethylthiazole; Bz, benzaldehyde; Pd, 2,3-pentanedione. N = 3. Error bars: SEM. [file Data_Sheet_1.pdf]

SUPPLEMENTAL INFORMATION

	Odor induced current			Wash induced current		
	Latency (ms)	Rise time (s)	Decay time (s)	Latency (ms)	Rise time (s)	Decay time (s)
AWC (TAX-2/TAX-4)	195.7±30.3	0.4±0.1	———	1252.3±353.4	1.4±0.3	9.9±1.3
ASH (OSM-9)	487.9±138.8	2±0.3	15.8±2.8	N.A.		

Table S1. Biophysical Properties of IAA-induced Currents from AWC and ASH Neurons. Related to Figure 1.

The rise times and decay times were obtained from 10% to 90% of the peak amplitude. Voltage: -70 mV. n ≥ 5.

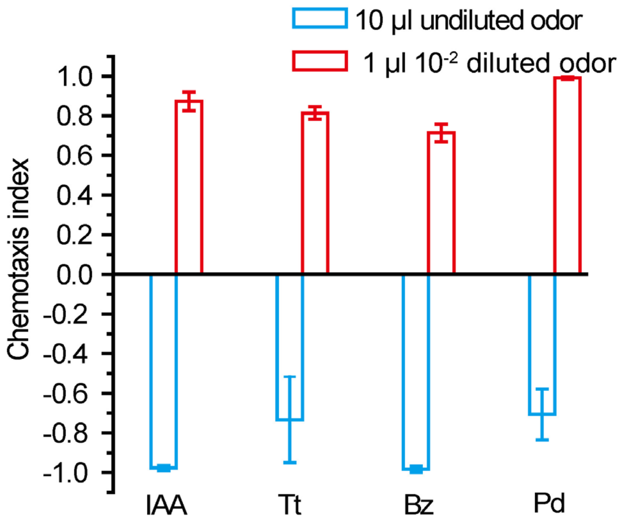

Figure S1. Odorants Which Are Attractive in Low Concentration Elicit Avoidance Response with High Concentration in *C. elegans*. Related to Figure 1.

Odorants were diluted with ethanol. IAA, isoamyl alcohol; Tt, 2,4,5-trimethylthiazole; Bz, benzaldehyde; Pd, 2,3-pentanedione. N = 3. Error bars: SEM.

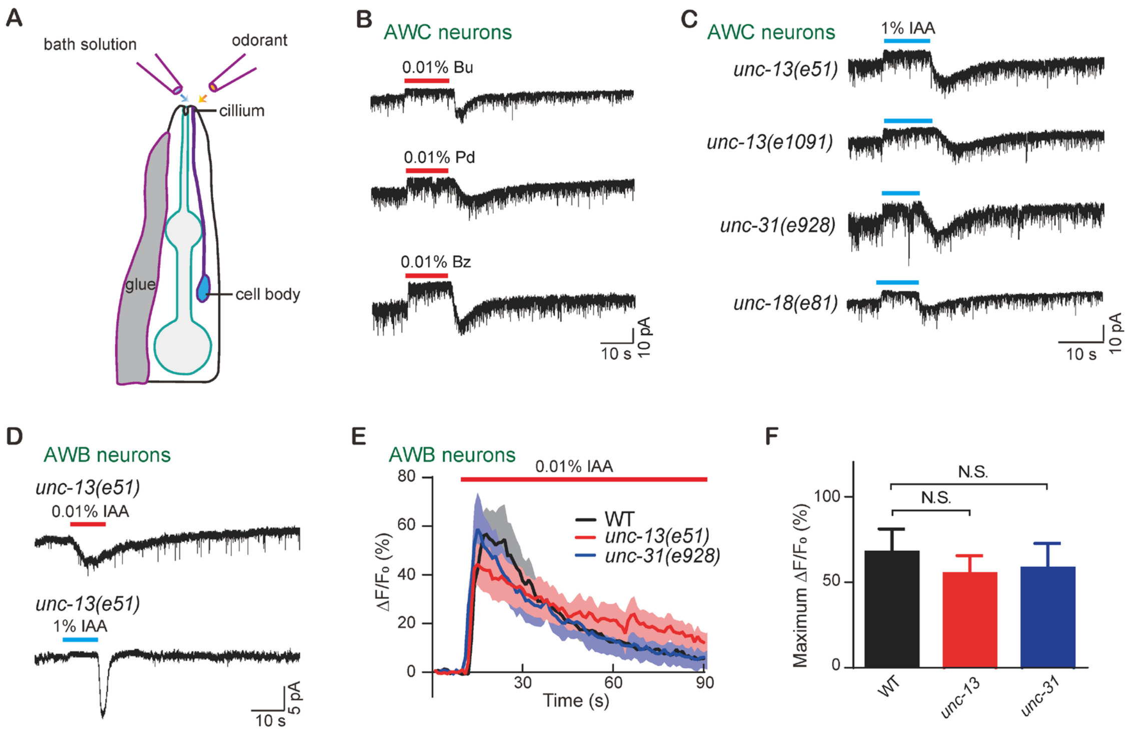

Figure S2. IAA induced Responses in AWC and AWB Neurons. Related to Figure 1.

(A) A schematic drawing of the calcium imaging and electrophysiological recording of odorant-induced responses in *C. elegans* olfactory neurons.

(B) Representative traces show olfactory currents from AWC neurons induced by 0.01% Bu (AWC^{ON}), 0.01% Bz (AWC^{ON} and AWC^{OFF}), and 0.01% Pd (AWC^{OFF}). Bz, benzaldehyde. Pd, 2,3-pentanedione. Bu, butanone.

(C) 1% IAA-induced currents in AWC neurons in *unc-13(e51), unc-13(e1091), unc-31(e928)* and *unc-18(e81)* mutants were equal to that in wild-type animals. $n \geq 4$.

(D) IAA-induced currents in AWB neurons in *unc-13* mutants. $n \geq 7$.

(E and F) 0.01% IAA-induced calcium responses in AWB neurons in *unc-13(e51)* and *unc-31(e928)* mutants. $n \geq 7$.

For patch-clamp recording, the cell membrane potential was voltage-clamped at -70 mV. Error bars: SEM. N.S.: not s
